# The Influence of Slit Lamp Shield Size and Design in Reducing Aerosol Transmission

**DOI:** 10.1167/tvst.10.13.33

**Published:** 2021-11-30

**Authors:** Mahmut Drogramaci, Mussa Adil Butt

**Affiliations:** 1 Princess Alexandra Hospital, Harlow, UK

**Keywords:** COVID-19, infections, aerosols, slit lamp, shield

## Abstract

**Purpose:**

Previous studies have highlighted the effectiveness of slit lamp shields in reducing aerosol spread. Our study investigated the optimal size and design for such shields.

**Methods:**

Two sets of shields were made; each set included five cardboards of the following dimensions: 1 (44 × 52 cm), 2 (44 × 44 cm), 3 (22 × 52 cm), 4 (22 × 33.5 cm), and 5 (44 × 22.5 cm). Cardboards in set 1 were kept flat whereas those in set 2 were curved using plastic frames. Aerosol was generated at the patient's position using a water spray bottle, and aerosol levels were measured at the face position of the examiner and on the slit lamp table using two GP2Y1014AU0F sensors. The measurements were recorded in particles/0.01f^3^ and analyzed using a Mann Whitney U test.

**Results:**

Mean background indoor aerosol was 559. After aerosol generation, the level increased to a mean of 571 in the absence of any kind of shield but to a mean of 567 when shields were in place (*P* < 0.05). Flat shield 1 provided the best protection against inhaled aerosol. Flat shield 2, despite its shorter height compared to shield 1, provided the best protection against precipitated aerosol on the table. Curving shield 5 significantly improved its protective properties against both inhaled and precipitated aerosol while keeping the short height that allowed better access during examinations.

**Conclusions:**

Shields reduced aerosol spread with curved shields being more effective while creating fewer physical restrictions. GP2Y1014AU0F particle sensors are effective tools for quantifying aerosol spread.

**Translational Relevance:**

An understanding of optimal slit lamp shield design will provide protection for examiners while facilitating effective examination.

## Introduction

There is a calculated 53,163,803 COVID-19 cases and 1,300,576 associated deaths globally as of November 3, 2020.^1^ Healthcare facilities are unfortunately an important source of viral transmission and therefore Public Health England, World Health Organization and Centers for Disease Control and Prevention have highlighted healthcare workers as high-risk individuals. Early in the pandemic, the World Health Organization began to recommend the use of personal protective equipment (PPE), such as gloves, masks, and gowns to prevent the spread of the virus.^2^

Ophthalmology practitioners, especially those performing slit lamp examinations, may be susceptible to infection because of proximity with the patient and the potential contamination of surrounding instruments and surfaces.^3^ The use of slit-lamp shields was recommended by the Royal College of Ophthalmologists and the American Academy of Ophthalmology in addition to other protective measures like face masks during slit lamp examination.^4^^,^^5^ However, there is no central guidance for the use of shields; therefore healthcare institutions are required to obtain their own, which may be of varying size and material.

Thanks to swiftly acting innovative companies, slit lamp shields became widely available in different sizes. It is conceivable that larger sized shields are likely to provide better protection, but they may also restrict operators from reaching their patients to perform certain tasks, for example, holding a patient's upper lid while performing a dilated fundal examination or reaching the joystick. In this study, we aimed to determine the optimal size and shape for a slit lamp shield that could provide the maximum protection without significantly restricting the examiner from reaching the patient.

## Methods

**Figure 1.**
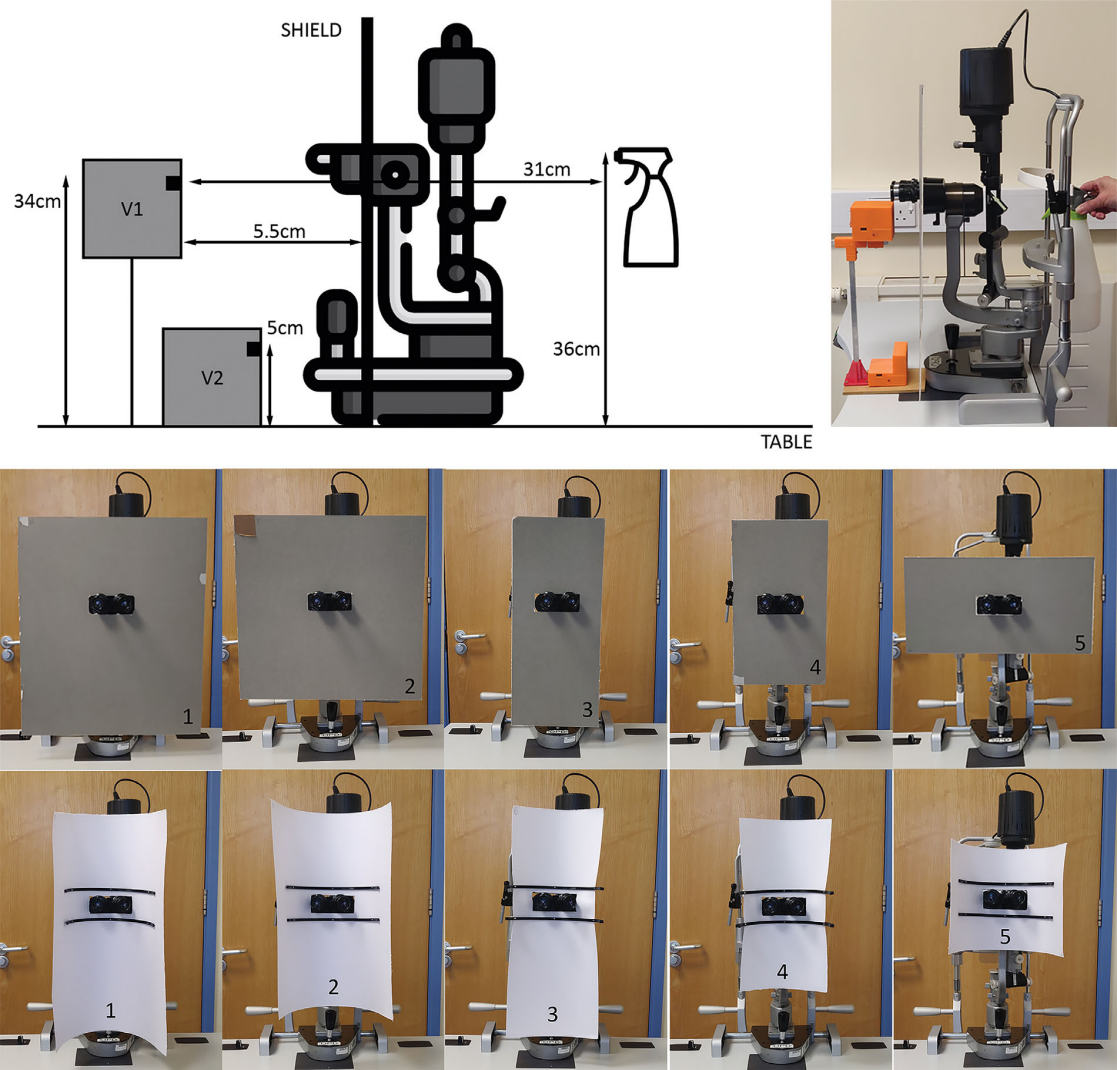
(a) Schematic of the experiment. (b) Photo of the experiment. (c–g) Flat shields 1 to 5 (left to right). (h–l) Curved shields 1 to 5 (left to right). Shield 1: 44 cm × 52 cm – Eye Piece located at 22 cm width and 35 cm height. Shield 2: 44 cm × 44 cm – Eye Piece located 22 cm width and 22 cm height. Shield 3: 22 cm × 52 cm – Eye Piece located 11 cm width and 35 cm height. Shield 4: 22 cm × 33.5 cm – Eye Piece located 11 cm width and 21.5 cm height. Shield 5: 44 cm × 22.5 cm – Eye Piece located 22 cm width and 13.5 cm height.

Two sets of cardboard made slit lamp shields were used in this study; each set included five shields of unique sizes (Fig. 1). The shields in set 1 were kept flat, whereas those in set 2 were bent around plastic semicircle frames of 180° and 200 mm in diameter. All shields had an 11 cm × 4.5 cm rectangle-cut hole to enable mounting them over the eyepiece of the slit lamp (Fig. 1).

A GP2Y1014AU0F particle matter sensor made by Sharp Corporation (Sakai, Osaka, Japan) was fitted into custom-built housing and used to measure aerosol concentration reaching the opposite side of slit lamp shields.^6^ The sensor counts particles with sizes that range from 0.5 to 2.5 µm. The data from the sensor was processed with a microprocessor and transferred to an android device through a Bluetooth connection. The data was received and analyzed in an android device using a purpose-built mobile application based on MIT App Inventor software.^7^ The number of particles was measured in particles/0.01f^3^. One sensor (V1) was fitted with a fan to produce airflow and simulate breathing in, the other (V2) did not include a fan and used to detect aerosol concentration that is likely to precipitate on slit lamp table.

Each sensor was calibrated for one minute before beginning the experiment for each shield. Aerosol generation was simulated using a spray bottle filled with water as used in previous studies.^8^^,^^9^ The nozzle was adjusted to create a mist and a single operator generated five sprays at two-second intervals, at the midpoint between headband and chin rest to maintain consistency (Fig. 1). Preliminary results were collected for an example of a shield currently being used by healthcare services.

Data was exported in Microsoft Excel in IBM Statistics for windows. Statistical analysis was completed using the Mann-Whitney U test, with statistical significance defined as *P* < 0.05.^10^^,^^11^

## Results

Our results showed that the mean indoor background aerosol detected in clinic settings by both sensors before applying any sprays was 559 particles/0.01f^3^. There was no statistically significant difference between the values detected by the two sensors. After applying five sprays of water at the patient's face position, mean aerosol values increased to 571 particles/0.01f^3^ in the absence of slit lamp shields and 567 particles/0.01f^3^ in the presence of slit lamp shields, the difference was statistically significant (*P* < 0.05).

Table 1 shows that curved shield 5 demonstrated the lowest mean aerosol in both sensors 1 and 2. Flat shield 1 in set 1, significantly reduced inhaled aerosol spread when compared to other shields in data from sensor 1 (*P* < 0.05). On the contrary, Flat shield 2 in set 1, despite its shorter height compared to shield 1, showed a greater reduction in precipitated aerosol spread when compared to all other flat shields in data from sensor 2 (*P* < 0.05). Table 2 shows mean values of aerosol, both inhaled by the examiner and precipitated on slit lamp table, for set 1 flat shields.

**Table 1.** Mean Aerosol Values for All Shields

		Sensor 1 (Inhaled)	Sensor 2
Shield Number	Geometry	Mean	Mean
1	Curved	577	575
	Flat	574	617
2	Curved	583	582
	Flat	583	574
3	Curved	583	590
	Flat	592	587
4	Curved	594	577
	Flat	586	598
5	Curved	574	560
	Flat	601	589

**Table 2.** Comparison of Flat Shields

	Sensor 1 (Inhaled)			Sensor 2			Both Sensors		
Shield Number	Mean	SD	Mann-Whitney U (*P* Value)	Mean	SD	Mann-Whitney U (*P* Value)	Mean	SD	Mann-Whitney U (*P* Value)
1	574	52	0	617	116	0	593	89	0.12
2	583	51		574	56		578	54	
1	574	52	0	617	116	0.01	593	89	0.27
3	592	59		587	69		590	64	
1	574	52	0.01	617	116	0.14	593	89	0.32
4	586	62		598	85		592	74	
1	574	52	0.01	617	116	0.10	593	89	0.32
5	601	87		589	64		595	77	
2	583	51	0.46	574	56	0.02	578	54	0.02
3	592	59		587	69		590	64	
2	583	51	0.88	574	56	0	578	54	0.02
4	586	62		598	85		592	74	
2	583	51	0.67	574	56	0.01	578	54	0.02
5	601	87		589	64		595	77	
3	592	59	0.51	587	69	0.49	590	64	0.96
4	586	62		598	85		592	74	
3	592	59	0.78	587	69	0.56	590	64	0.85
5	601	87		589	64		595	77	
4	586	62	0.50	598	85	0.85	592	74	0.83
5	601	87		589	64		595	77	

Curved shield 1 in set 2 also significantly reduced aerosol spread when compared to curved shields 2, 3, and 4 in data from sensor 1 (*P* < 0.05). But curving shield 5 enhanced its protection both against inhaled and precipitated aerosols significantly (*P* < 0.05). Data from sensor 1 showed that curved shield 5 provided a statistically significant reduction in inhaled aerosol compared to curved shields 2 and 4 and statistically nonsignificant reduction compared to curved shields 1 and 3. Data from sensor 2 also showed statistically significant superiority of curved shield 5 over all other curved shields (*P* < 0.05) while keeping its advantage of short height that posed fewer restrictions on accessibility to the patient during an examination. Table 3 shows mean values of aerosol, both inhaled by the examiner and precipitated on slit lamp table, for set 2 curved shields.

**Table 3.** Comparison of Curved Shields

	Sensor 1 (Inhaled)			Sensor 2			Both Sensors		
Shield Number	Mean	SD	Mann-Whitney U (*P* Value)	Mean	SD	Mann-Whitney U (*P* Value)	Mean	SD	Mann-Whitney U (*P* Value)
1	577	59	0.002	575	54	0.034	576	57	0
2	583	52		582	56		583	54	
1	577	59	0.038	575	54	0.003	576	57	0
3	583	62		590	67		586	65	
1	577	59	0	575	54	0.988	576	57	0.01
4	594	57		577	60		583	59	
1	577	59	0.546	575	54	0	576	57	0
5	574	44		560	51		567	48	
2	583	52	0.386	582	56	0.416	583	54	0.96
3	583	62		590	67		586	65	
2	583	52	0.053	582	56	0.056	583	54	0.01
4	594	57		577	60		577	60	
2	583	52	0.012	582	56	0	583	54	0
5	574	44		560	51		567	48	
3	583	62	0.009	590	67	0.007	586	65	0.01
4	594	57		577	60		577	60	
3	583	62	0.131	590	67	0	586	65	0
5	574	44		560	51		567	48	
4	594	57	0	577	60	0	577	60	0.01
5	574	44		560	51		567	48	

Mean inhaled and precipitated aerosol levels detected from both sensors showed statistically significant superiority of curving the shields around the examiners face. Although this superiority was more prominent for precipitated aerosol detected by sensor 2 for shields 1, 3, 4, and 5 and for inhaled aerosol detected by sensor 1 for shield number 5. Surprisingly, data from sensor 2 also showed that the flat version of shield 2 provided better protection against precipitated aerosol compared to its curved version. Table 4 shows mean values of aerosol, both inhaled by the examiner and precipitated on the slit lamp table, for curved and flat shields.

**Table 4.** Comparison of Flat Versus Curved Shields

		Sensor 1 (Inhaled)			Sensor 2			Both Sensors		
Shield Number	Geometry	Mean	SD	Mann-Whitney U (*P* Value)	Mean	SD	Mann-Whitney U (*P* Value)	Mean	SD	Mann-Whitney U (*P* Value)
1	Curved	577	59	0.905	575	54	0	576	2	0
	Flat	574	52		617	116		593	3	
2	Curved	583	52	0.701	582	56	0.01	576	2	0.06
	Flat	583	51		574	56		593	3	
3	Curved	583	62	0.072	590	67	0.44	586	3	0.53
	Flat	592	59		587	69		590	3	
4	Curved	594	57	0.172	577	60	0.02	586	3	0
	Flat	586	62		598	85		590	3	
5	Curved	574	44	0.012	560	51	0	567	2	0
	Flat	601	87		589	64		595	4	

## Discussion

Previous studies confirmed the effectiveness of slit lamp shield use in reducing aerosol transmission; however, the use of a slit lamp is a technical skill that requires fine adjustments, and therefore ease of access to the controls is essential. While larger shields are more likely to provide better protection, they could also cause difficulty for the examiner to access his or her patient and slit lamp parts during an examination, therefore it could impede effective examination. Alternatively, small shields may enable ease of control but may not offer enough protection. Aerosol spread is usually detected using droplet imaging systems or light scattering technology. Droplet imaging uses fluorescein and photographic imaging under ultraviolet light to trace aerosol droplets^12^; although inexpensive, this methodology does not enable quantitative analysis of the data and therefore cannot be used to show a statistically significant difference between different types of slit lamp shields. Alternatively, the light scattering method enables statistical comparison; however, it usually entails the use of expensive and bulky aerosol detectors that may be difficult to use in slit lamp settings.^13^ In our study, we used two custom-built aerosol detectors to detect inhaled aerosols at examiners face position and precipitated aerosols on slit lamp table. We investigated the optimal size and design of a slit lamp shield that could provide the best protection against both inhaled and precipitated aerosol.^13^^,^^14^

Our results show that the use of slit lamp shields is effective at reducing the aerosol spread and that increasing the size of the shields increases their efficacy. Out of the 5 different sizes of slit lamp shields, shield 1 was the largest and the most effective. Such results are expected as a larger shield would provide larger screens against which aerosol particle would bounce back. But equally a larger shield would add more restrictions in reaching the patient and slit lamp joystick. Khadia et al.^15^ attempted to partly overcome the restrictions in reaching patients imposed by the use of large barriers by creating holes in the bottom of the shield to allow room for the examiner's hands.

**Figure 2.**
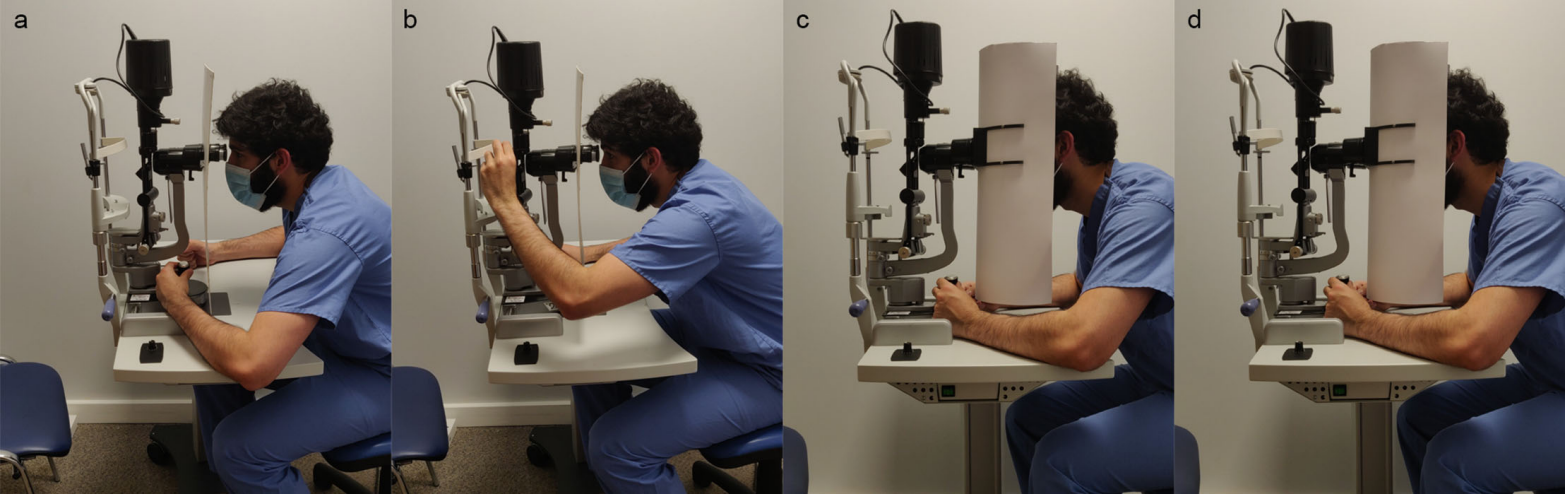
Use of flat and curved shields. (a, b) Use of a flat shield causing restriction for the examiner. (c, d) Use of curved shields with reduced restriction.

In our study, we aimed to increase the efficacy of slit lamp shields without adding further restriction to accessibility by bending the shields around plastic frames with a 20 cm arc and creating curved shields to reduce their overall width. The results showed that curving the shields not only improves accessibility but also provides better overall protection. Figure 2 illustrates that flat shield 1 requires the examiner to bend an arm around the width of the shield to access the joystick. However, by curving shield 1, the need for this is significantly reduced, and the examiner is not stretching to reach the controls. The values in Table 3 show that curving the shields does not only improve accessibility but also provide better protection.

It is logical to think that a shield with greater height and width, and therefore a greater surface area, would provide better protection against both inhaled and precipitated aerosol. This effect can be seen when comparing data from sensor 1 for both curved and flat shields 1 and 2. However, data from sensor 2 shows that flat shield 2, despite of its shorter height compared to flat shield 1, provided greater protection against the precipitated aerosol spread (*P* < 0.05). This may be explained by a mechanism of droplets deflecting off the shield and further studies into the differences of this phenomenon between flat and curved shields are required. Similarly, curved shield 5, despite its shorter height compared to all other shields, provided the best protection against both inhaled and precipitated aerosol. This is possibly due to the fact that the slit lamp parts that fall between the patient and the examiner change the aerodynamics of aerosol travel, and minimize the advantages of shields with larger heights. Therefore we can propose that curved shields with large width but smaller heights similar to shield 5 in set 2 of our experiment has benefits for both ease of use for the examiner and protection from aerosol.

One of the limitations of our study is that our measurements were restricted to aerosols of 2.5 µm in size because of the built-in specification of our chosen sensors. Aerosols ranging in size from 1.0 to 5.0 µm generally remain in the air, whereas larger particles are deposited on surfaces^16^; therefore we believe that monitoring aerosols of 2.5 µm in size is a good indicator for the efficacy of slit lamp shields. Similarly, the design of the sensor 1 was chosen to simulate the mechanism on inhalation in the human body. However, we cannot accurately replicate all factors that influence the biomechanics of inhalation. Nonetheless, we believe that the gross result of manipulating shield size and design on aerosol reduction is a valuable outcome from this study.

Aerosol distribution is dependent on the airflow within a consultation room. This factor can vary depending on location and arrangement in a consultation room. Therefore the outcomes of our study may not be generalizable to other settings. We chose to conduct five sprays for each shield and calibrated our sensors to collect two measurements per second during the recording phase to enhance the accuracy of our calculated mean aerosol per shield. However, we only conducted one round of testing for each shield and with further testing this would have added greater accuracy to our results.

The mechanism to explain the equal, if not greater, protection provided by curved shields compared to flat shields is beyond the scope of this study. It may be related to the effect of shield design on aerosol trajectory and is something we believe should be investigated further. Even so, we believe that the ease of examination that curved shields provide is their main advantage over flat shields.

## Conclusion

To conclude, the use of a slit lamp shield is effective in reducing aerosol exposure during an examination. Curved shields are superior for ease of use for the operator and reduction of aerosol spread. We recommend the use of curved shields for all slit lamp operators during the current pandemic. A GP2Y1014AU0F particle matter sensor is an effective, reliable, standalone and cheaper approach to quantify aerosol and could be used to study the efficacy of different types of personal protective measures.
